# Primary Rosai-Dorfman Disease of the Proximal Radius Masquerading as Tuberculosis of Elbow

**DOI:** 10.7759/cureus.7858

**Published:** 2020-04-27

**Authors:** Balaji Zacharia, Karthikeyan Manicam

**Affiliations:** 1 Orthopaedics, Government Medical College, Kozhikkode, IND

**Keywords:** tuberculosis, elbow, rosai dorfman disease

## Abstract

A 36-year-old female presented with pain and progressive decrease in the range of movements in the left elbow. There was contact with an open case of pulmonary tuberculosis. There were no constitutional symptoms except fever. X-ray showed periarticular osteopenia with destruction of the radial neck and head. MRI scan findings were also consistent with tuberculosis of the elbow. Histopathology examination confirmed the diagnosis of Rosai-Dorfman disease. The patient was treated with glucocorticoid and methotrexate and was asymptomatic after 18 months.

## Introduction

Rosai-Dorfman disease (RDD) is also known as sinus histiocytosis with massive lymphadenopathy (SHML). It is a rare benign disorder of histiocytic proliferation of unknown etiology [1]. Sinus histiocytosis is another name for this disorder. It is due to an overproduction of monocytes [2]. The disease has a predilection to affect lymph nodes in adolescent children and young adults. Extra-nodal involvement can occur in 28% to 43% of cases. Skin, eyes, bones, nasal sinuses, central nervous system, salivary glands, kidneys, respiratory tract, liver, breast, and gastrointestinal tract are the most common extra-nodal sites [3]. The inflammatory or hyperplastic variety of RDD seen in a lymph node can have a spontaneous regression, whereas extra-nodal disease can recur after many years [4]. There is an association of RDD with autoimmune diseases [5].

There were reports of RDD involving the bone. The wrist, tibia, femur, clavicle, skull, maxilla, calcaneus, phalanx, metacarpals, and sacrum are some of the areas where RDD has been reported [6,7]. In this report, we describe a case of RDD of the left elbow.

## Case presentation

A 36-year-old female presented with dull aching pain in the left elbow for eight months. She noticed a progressive loss in the range of movements of her left elbow. One of her close relatives was on anti-tubercular treatment for pulmonary tuberculosis. She had a low-grade fever without cough, chills, rigor, night sweats, and loss of appetite or loss of weight. At the time of examination, there was fullness around the lateral aspect of the elbow, with synovial thickening around the radio-capitular joint. There was tenderness over radial head. The range of flexion was 70 degrees, and the supination and pronation were restricted to 30 degrees.

**Figure 1:**
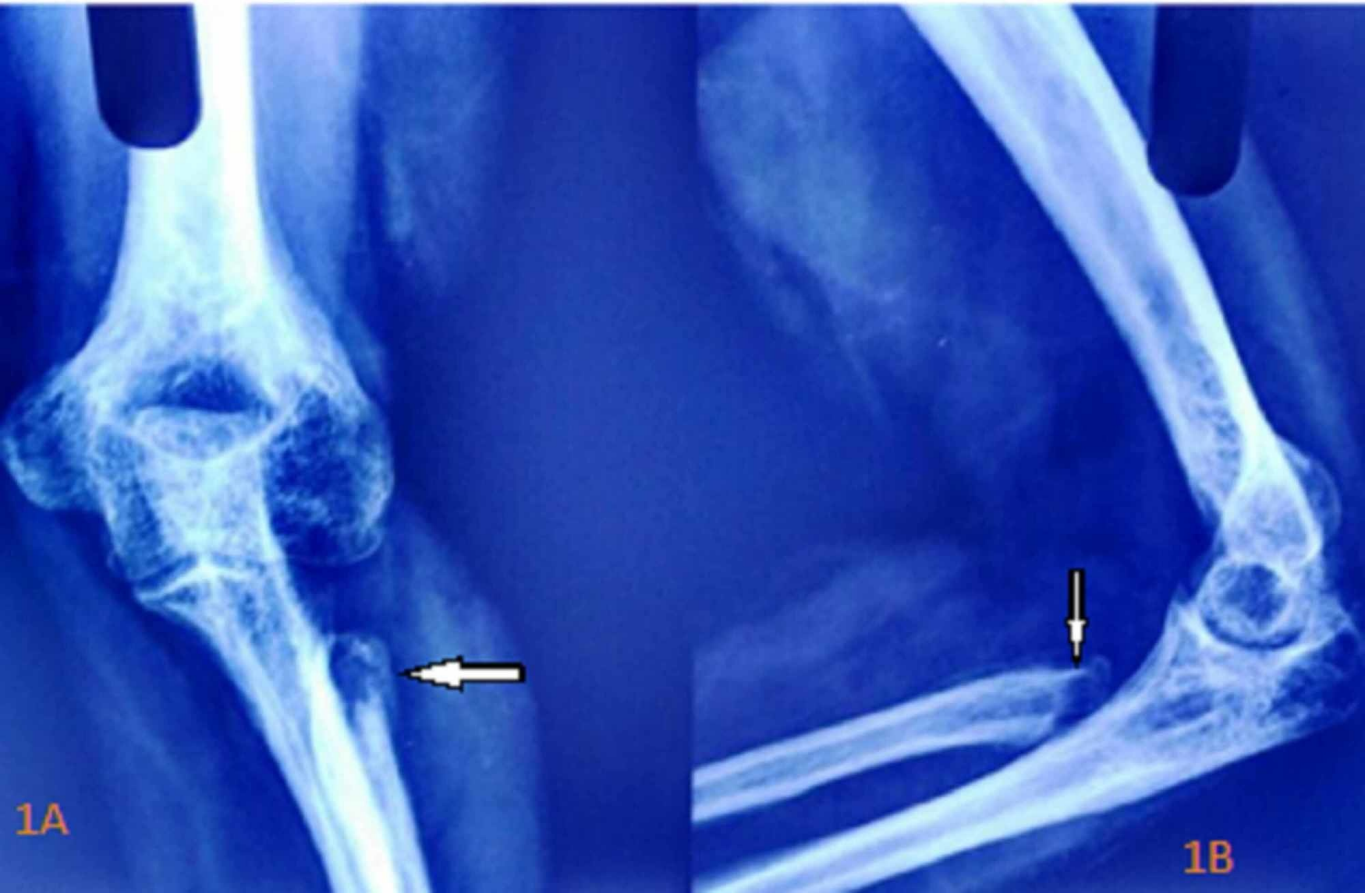
X-ray of the left elbow Anteroposterior (1A) and lateral (1B) view showing generalized osteopenia in the lower end of the humerus and upper end of the ulna and destruction of head and neck of the radius with periosteal reaction distal to destruction. The distal humerus and trochlea-humeral joint are intact.

**Figure 2:**
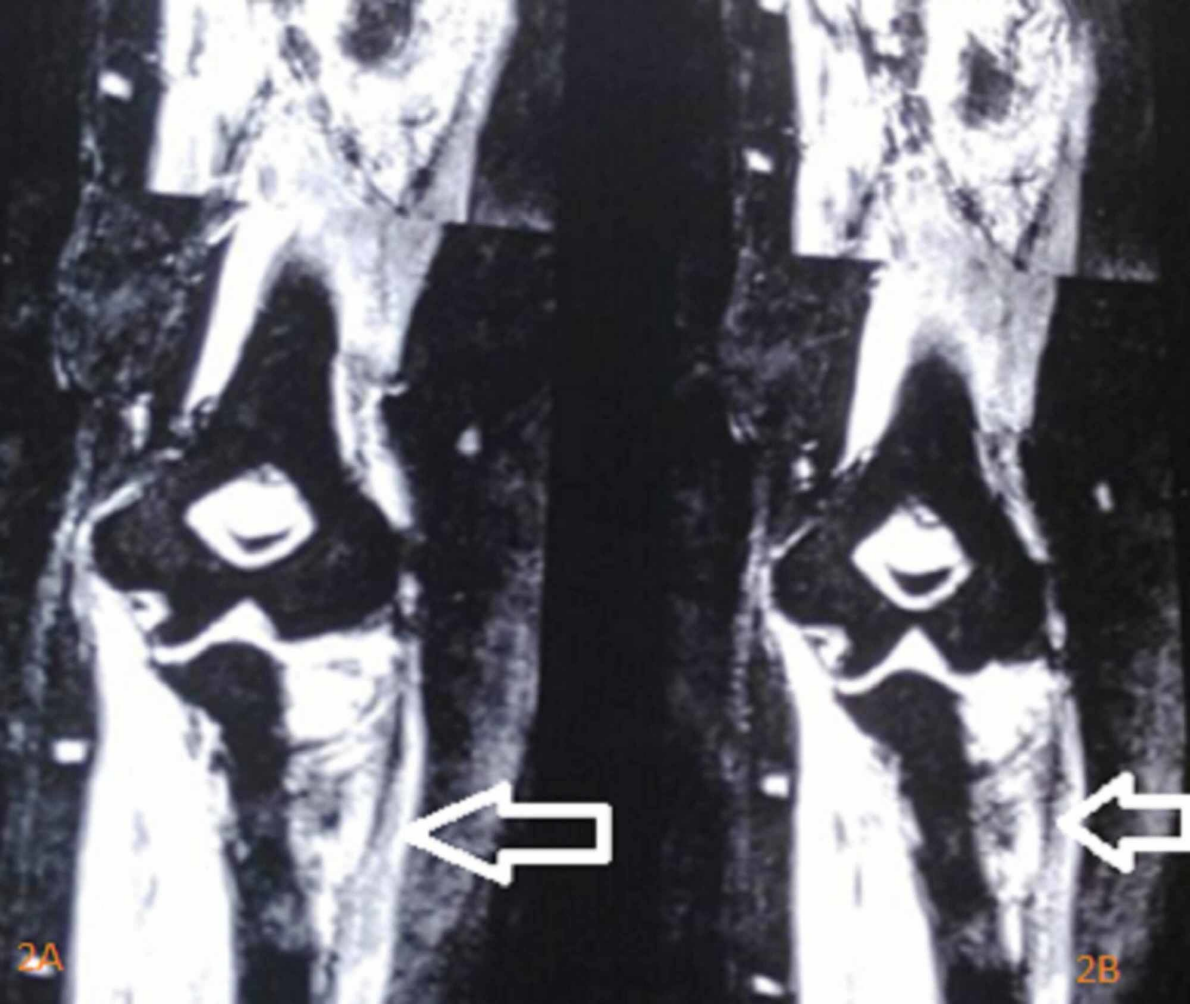
MRI scans of the left elbow MRI scans of the left elbow showing a 4.5 × 3.5 × 2 cm soft tissue lesion in the anterior part of the proximal radius with erosion of the anterior part of the head and neck of the radius. The lesion is hyperintense on T2 fat-suppressed view. Periosteal reaction is seen in the neck of the radius. Effusion and synovial thickening are suggestive of tuberculosis of the elbow.

Hemogram showed a total leukocyte count of 12,000/mm^3^ with increased neutrophil count, hemoglobin of 12 g/dL, and hematocrit of 42%. Renal and liver function tests were all within normal limits. The erythrocyte sedimentation rate (ESR) was elevated to 70 mm per hour. The C-reactive protein was within normal limits. Mantoux test was positive. Sputum culture and GeneXpert® test (Cepheid, Sunnyvale, CA, USA) were negative. Fungal culture and real-time polymerase chain reaction were negative. Plain X-ray of the left elbow showed peri-articular osteoporosis with the destruction of the head and neck region of the proximal radius. Even though there was severe osteopenia, there was no destruction in the distal articular surface of the humerus and trochlea-humeral joint. Periosteal reaction was seen beyond the destruction in radius (Figures 1A, 1B). MRI revealed 4.5 × 3.5 × 2 cm soft tissue lesion in the anterior part of the proximal radius. There was erosion of the anterior part of the head and neck of the radius. Periosteal reaction was seen around the neck. Effusion and synovial thickening were seen in the elbow joint. MRI features were suggestive of tuberculosis of the elbow (Figure 2A, 2B). 

With the above clinical features and investigations, our diagnosis was tuberculosis of the elbow. Pigmented villonodular synovitis, early synovial osteochondromatosis, and inflammatory arthritis were considered as the differential diagnosis.

**Figure 3:**
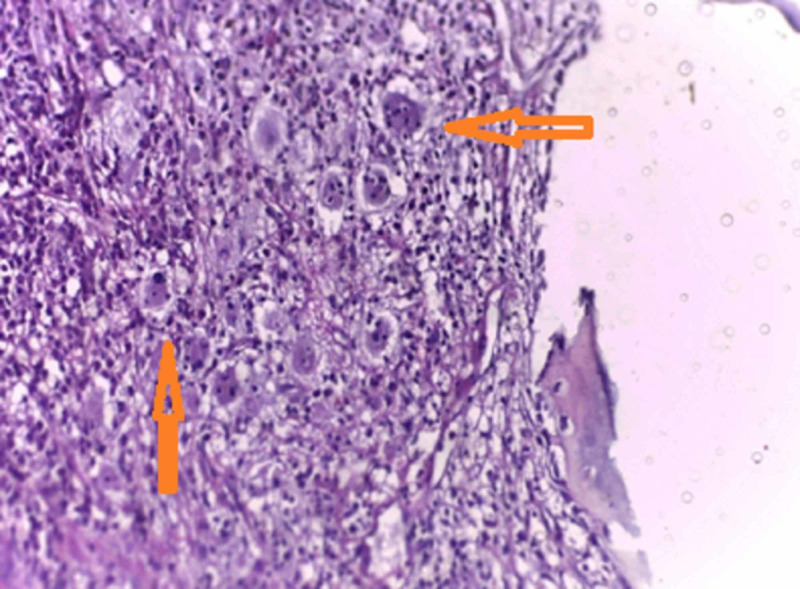
Histology showing emperipolesis Histopathology specimen from open biopsy showing a collection of inflammatory cells with histiocytes and marked emperipolesis (engulfment of intact lymphocytes within the cytoplasm, arrowheads).

**Figure 4:**
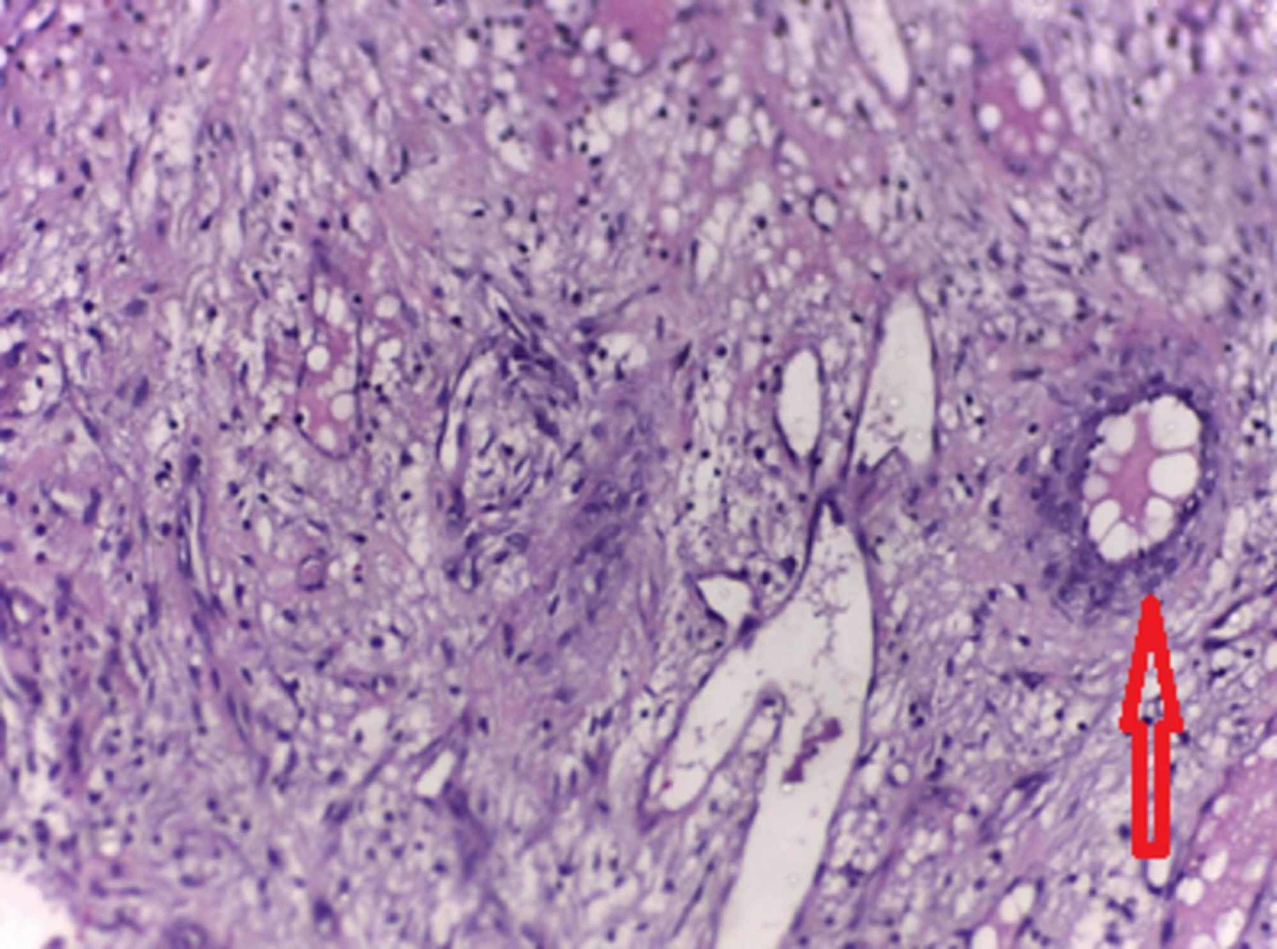
Histology showing palisaded granuloma Histopathology specimen showing foci of necrosis with palisaded granuloma (arrowheads).

**Figure 5:**
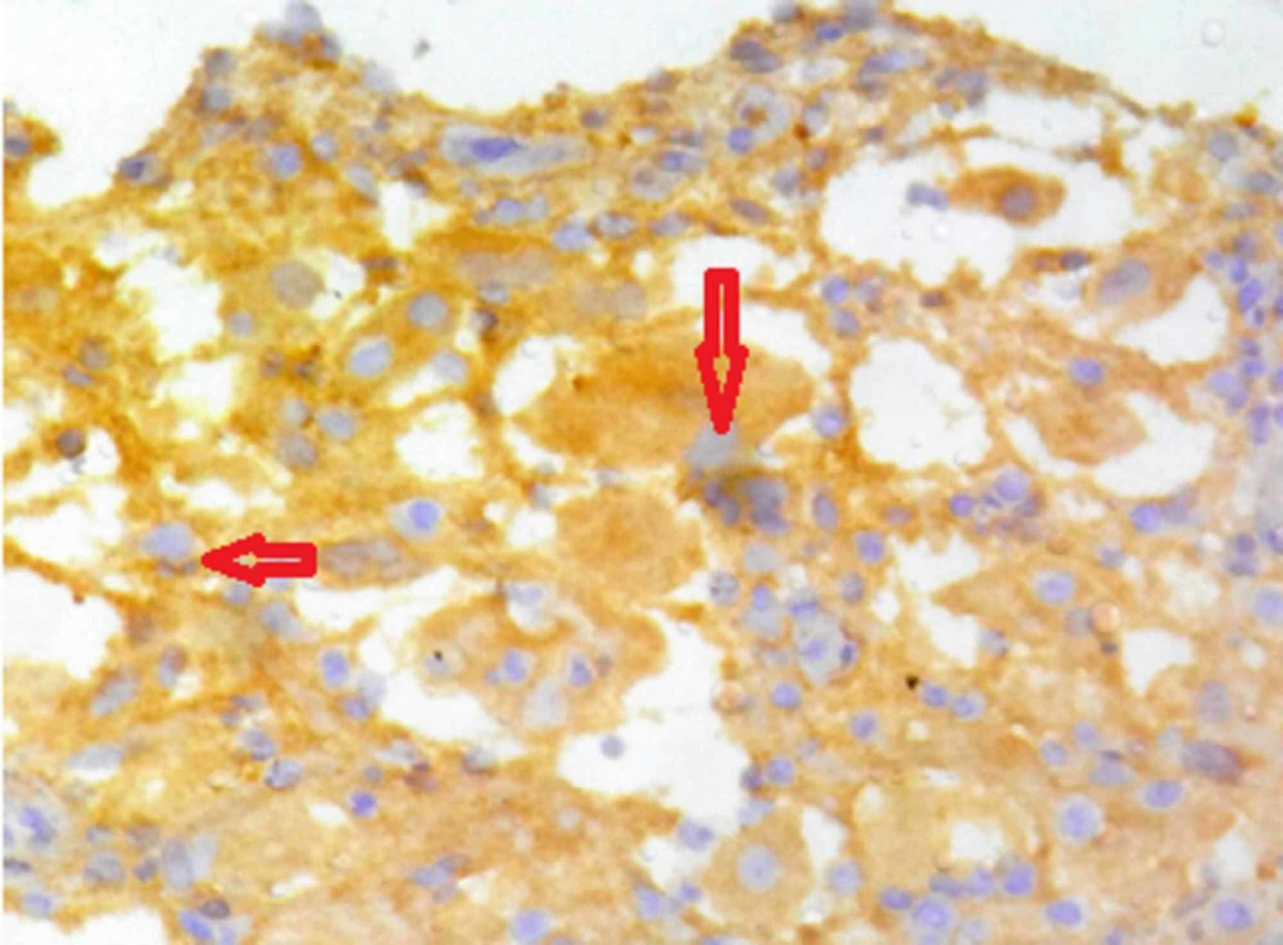
S-100 positive histiocytes Immunohistochemistry of the histiocytes in the lesion showing positivity for S-100 protein, confirming the diagnosis of Rosai-Dorfman disease (arrowheads).

An open biopsy was performed through a lateral approach. Grossly, there were multiple bits of bone and synovial tissue measuring 3 × 2 × 1 cm. Histopathology showed a collection of chronic inflammatory cells in the bone and soft tissues with histiocytes showing marked emperipolesis and foci of necrosis with palisaded granuloma (Figures 3, 4). Immunohistochemistry of the histiocytes in the lesion showed positivity for S-100 protein (Figure 5), confirming the diagnosis of solitary primary Rosai-Dorfman disease of the upper end of the radius.

Post-operatively the left upper limb was immobilized for about two weeks in a long arm slab. The patient was further followed up in the oncology department. She was treated with glucocorticoid and methotrexate. After 18 months, she is asymptomatic, even though there is a restriction of elbow movements. There is no evidence of recurrence.

## Discussion

RDD or SHML is a rare benign disease of unknown etiology, which presents with cervical lymphadenopathy. It is usually seen in younger patients [8]. The form affects various regions of the soft tissue, skin, upper respiratory tract, gastrointestinal tract, breast, bones, and the central nervous system, and is more common in patients with immune abnormalities [9]. Around 5% of cases involve the bone; they are usually associated with extraneous manifestations [10]. Primary solitary osseous involvement is very uncommon. RDD appears as lytic lesions [7]. Clinically, they may be confused with and inflammatory conditions. The differential diagnosis of RDD includes lymphoma, malignant histiocytosis, disseminated tuberculosis, and Langerhans cell histiocytosis (LCH). The phenomenon of is central in differentiating RDD. The presence of weight loss, night sweats, and malignant cells staining positive for CD45 favors the diagnosis of lymphoma. Malignant histiocytosis differs from RDD clinically by its rapid downhill course and pathologically by the presence of malignant having bizarre, pleomorphic nuclei. The histiocytes in LCH have a characteristic folded and grooved nucleus and exhibit CD1a positivity. Disseminated tuberculosis can be ruled out based on the absence of and negative staining for acid-fast bacilli by Ziehl-Neelsen stain [11].

In the majority of the cases, RDD runs a benign self-limiting course, and no treatment is necessary. However, in patients with massive nodal or extra-nodal involvement with threatening organ dysfunction, therapy is indicated [11,12]. For those who require therapy due to persistent or worsening symptoms, treatment modalities include surgery, chemotherapy, radiotherapy, and steroids [13].

In our case, there was a history of contact with open pulmonary tuberculosis. The clinical and radiological features are consistent with tuberculosis of the elbow. Our region is an endemic area of tuberculosis. Hence, we considered the diagnosis of tuberculosis of the elbow joint, but the biopsy proved it to be a case of RDD.

## Conclusions

This is a rare case of isolated osseous involvement of RDD of the left proximal radius. The clinical presentation causes some challenges in diagnosis.
